# Adherence to a healthy sleep pattern is associated with lower risks of incident falls and fractures during aging

**DOI:** 10.3389/fimmu.2023.1234102

**Published:** 2023-08-17

**Authors:** Tao Zhou, Xue Dai, Yu Yuan, Qiaochu Xue, Xiang Li, Mengying Wang, Hao Ma, Yoriko Heianza, Lu Qi

**Affiliations:** ^1^ Department of Epidemiology, School of Public Health (Shenzhen), Sun Yat-sen University, Shenzhen, China; ^2^ Department of Epidemiology, School of Public Health and Tropical Medicine, Tulane University, New Orleans, LA, United States; ^3^ Department of Occupational and Environmental Health, Key Laboratory of Environment and Health, Ministry of Education and State Key Laboratory of Environmental Health (Incubating), School of Public Health, Tongji Medical College, Huazhong University of Science and Technology, Wuhan, China; ^4^ Department of Epidemiology and Biostatistics, School of Public Health, Peking University Health Science Center, Beijing, China; ^5^ Department of Nutrition, Harvard T.H. Chan School of Public Health, Boston, MA, United States

**Keywords:** sleep, sleep patterns, bone mineral density, fall, fracture

## Abstract

**Background:**

Autoimmune diseases are more common among people with unhealthy sleep behaviors, and these conditions have been linked to aging-related bone health. However, there have been few studies that examined the correlation between recently developed sleep patterns based on sleep duration, sleepiness, chronotype, snoring, insomnia, and the incidence of falls and fractures.

**Methods:**

We used a newly developed sleep pattern with components of sleep 7 to 8 h per day, absence of frequent excessive daytime sleepiness, early chronotype, no snoring, and no frequent insomnia as healthy factors to study their relationship with the incidence of falls and fractures. The analysis was conducted among 289,000 participants from the UK Biobank.

**Results:**

The mean follow-up period was 12.3 years (3.5 million person-years of follow-up), and 12,967 cases of falls and 16,121 cases of all fractures were documented. Compared to participants exhibiting an unfavorable sleep pattern, those adhering to a healthy sleep pattern experienced a 17% and 28% reduction in the risks of incident falls (hazard ratio [HR], 0.83; 95% CI, 0.74–0.93) and all fractures (HR, 0.72; 95% CI, 0.66–0.79) during follow-up. In addition, participants exhibiting a healthy sleep pattern, together with a high genetically determined bone mineral density (BMD), showed the lowest risks of falls and fractures.

**Conclusion:**

A healthy sleep pattern was significantly linked to decreased risks of incident falls and fractures. The protective association was not modified by genetically determined BMD.

## Introduction

Age-related loss in bone mineral density (BMD), falls, and the occurrence of fractures related to osteoporosis are among the primary causes for disability in the elderly population and have become an important public health concern arising from the increasing size of the aging population (1). Decreased bone density and strength resulting from low bone mass make it a significant risk factor for fractures, and genetic influences on fracture all act through BMD (2). In the elderly population, falls are responsible for 87% of all fractures. These fractures predominantly occur as a result of low-impact injuries in osteoporotic bones (3).

Examination and identification of the modifiable risk factors are important in preventing falls and fractures. Recently emerging evidence indicates that sleep behaviors may have a critical role in regulating bone health, falls, and fractures (4–8). Unhealthy sleep behaviors result in the elevation of several autoimmune-related proinflammatory cytokines (9), which present promising targets for enhancing the treatment and diagnosis of inflammatory bone loss (10, 11). A recent study showed that the systemic immune-inflammation index could predict the risk of low BMD or osteoporosis in postmenopausal women (12).

In epidemiological studies, several sleep disorders or sleep behaviors have been related to falls and fractures. For example, long (≥10 h) or short (≤5 h) sleep, daily napping, insomnia, and poor sleep quality have been directly related to greater risks of falls and fractures among older populations (8, 13–15). The observed associations may be attributed to impaired bone health since short or excessive sleep duration (6), daytime napping (5, 16), and later sleep (17) are also related to low BMD (4). However, these previous studies only analyzed individual sleep behaviors separately. Of note, sleep traits are highly correlated (18), and the change of one behavior may trigger a change of others (19). To better characterize the joint impact of the various sleep behaviors, we newly established sleep patterns, incorporating sleep duration, insomnia, dozing, nap, and chronotype, which have been validated and associated with decreased risks of cardiovascular diseases in two independent cohorts (19–21). However, no research has examined the relationships between sleep patterns and the incidence of falls and fractures in prospective settings (19).

In the present study, we conducted a prospective analysis to investigate whether our newly generated sleep patterns were associated with the subsequent incidence of falls and fractures, as well as BMD among 289,000 participants from the UK Biobank. We particularly assessed whether such associations were modified or mediated by immune factors.

## Methods

### Research design

This study was based on participants of the UK Biobank, which is a prospective study cohort including 0.5 million participants aged 40 to 69 years (22). After excluding non-British participants (*n* = 59,919), participants who had a mismatch between their self-reported sex and genetic sex (*n* = 334), those with a history of fracture at baseline (*n* = 48,490), those without information on sleep behaviors (*n* = 68,894), and those without information on falls, fractures, and covariates including age, sex, and BMI (*n* = 35,869) from the full dataset, the final analysis included 289,000 participants.

### Evaluation of sleep behaviors

Sleep behaviors as reported by the participants, including sleep duration, daytime sleepiness, chronotype, snoring, and insomnia, were used to construct a healthy sleep score, which was in accordance with our previous study (21). In brief, we defined low-risk sleep factors as a sleep duration of 7 to 8 h, no frequent daytime sleepiness, early chronotype, no snoring, and no insomnia. For each of the five sleep components, participants who meet the criteria received 1 point or 0 otherwise, and the sum is a healthy sleep score that ranged from 0 to 5. A higher healthy sleep score indicated a healthier sleep pattern. We divided the score into three subgroups for further analyses: favorable (score 4–5), intermediate (score 2–3), and unfavorable (score 0–1).

### Ascertainment of outcome

The primary outcome was incidence falls and all fractures and hip fracture during follow-up. ICD-10 codes used in medical records to document the cases of fractures can be found in **Supplementary Table 1** (23). Incident falls were defined using ICD code W00-W19. BMD (eBMD) derived from heel ultrasound was assessed with a Sahara Clinical Bone Sonometer (Hologic Corporation, Bedford, MA, USA), which measured quantitative ultrasound parameters including the speed of sound as well as broadband ultrasound attenuation. DXA-BMD (total BMD) was acquired using an iDXA instrument (GE-Lunar, Madison, WI, USA), and the method is provided by the UK Biobank official website.

### Covariates assessment

At the assessment centers, a touchscreen questionnaire was administered to gather baseline data, such as age, sex, smoking status (never, previous, or current), and drinking status (never, previous, or current). Height and weight were examined during the initial visit. BMI was calculated as weight(kg)/height(m)^2^. We determined physical activity (MET-minutes/week) by calculating the time spent walking and doing moderate or vigorous physical activities, taking into account the varying levels of intensity involved in different physical activities (24). We imputed missing data of MET-minutes per week using a mean value. Serum vitamin D level was measured using the method of Chemiluminescent Immunoassay-direct competitive (Diasorin S.p.A). Participants who responded “Prefer not to answer” were excluded from the analysis. Blood cell counts, including white blood cells (WBCs), neutrophils, monocytes, and lymphocytes, were performed using an LH750 hematology analyzer (Coulter, Beckman Coulter, Brea, CA, USA). Serum C-reactive protein (CRP) was measured using a Beckman Coulter clinical chemistry analyzer [AU5800 Immuno-turbidimetric, Beckman Coulter (UK)].

### Genotyping and polygenic scores

The UK Biobank provided the information about genotyping, imputation, and quality control of the single-nucleotide polymorphisms (SNPs). A weighted polygenic score (PGS) was calculated using the equation: weighted PGS = (β_1_ × SNP_1_ +…+β_n_ × SNP_n_) × (number/sum of the β coefficients), where “SNP” represented the risk allele number for each SNP and the β coefficients were based on a GWAS study, in which genetic variants were identified for BMD estimated by heel quantitative ultrasound (23). All SNPs used to calculate the PGS follow the Hardy–Weinberg equilibrium with a *p*-value greater than 1E-12 among the white British population. The PGS explained 16% of the variance of eBMD.

### Statistical analysis

Multivariable Cox proportional hazard models were applied to examine the association of sleep-related exposures with incident all fractures and hip fracture, while adjusting for covariates including age, sex, BMI, assessment centers, smoking status (never, previous, or current), drinking status (never, previous, or current), serum vitamin D levels, and physical activity (MET-minutes/week). The proportional hazards assumption was assessed by conducting the Wald test for the interaction term between time and exposure. A *p*-value for interaction less than 0.05 was considered as a violation of the assumption. No violation of the assumption was found. We used linear regression to explore the association between the healthy sleep pattern and BMD, adjusting for age, sex, BMI, assessment centers, alcohol intake and smoking status, serum vitamin D levels, and physical activity. To determine whether BMD PGS and sex would modify the association between a healthy sleep pattern and falls, fractures, and BMD, we introduced an interaction term consisting of healthy sleep score and BMD PGS/sex/immune parameters in the models individually. Mediation analyses were used to estimate the extent to which immune factors may explain this relationship between sleep score and BMD. To address the issue of reversed causality, we performed sensitivity analyses by excluding incident cases during the initial 2 years of follow-up. We also conducted sensitivity analysis by further adjusting for menopausal status when testing the association of sleep with BMD, falls, and fractures among women. All data were were analyzed using SAS 9.4 for Windows (SAS Institute Inc, Cary, NC). A two-tailed *p* < 0.05 was considered statistically significant.

## Results

A total of 289,000 participants were included in the study, and 155,810 (54%) were women. The mean (SD) age and BMI was 57 (8) years and 27 (5) kg/m^2^, respectively. During a mean 12.3 years of follow-up, providing 3.5 million person-years of follow-up, 12,967 cases for falls, 16,121 for all fractures, and 2,655 for hip fracture were documented. Women were more likely to have incident falls, all fractures, and hip fracture compared to men (all *p* < 0.001). The mean (SD) eBMD and DXA-BMD was 0.547 (0.135) and 1.203 (0.150) g/cm^2^, respectively, and men showed higher eBMD and DXA-BMD compared to women (0.521 vs. 0.576 g/cm^2^, *p* < 0.001; 1.1164 vs. 1.2945, *p* < 0.001). Men showed higher levels of WBC, monocytes, and neutrophils, but lower level of lymphocytes and CRP (all *p* < 0.001) compared with women (**Table 1**).

**Table 1:** Baseline characteristics of UK Biobank participants.

	All	Women	Men
*N*	289,000	155,810	133,190
Age, years	56.7 ± 8.0	56.1 ± 8.0	57.3 ± 8.0
BMI, kg/m^2^	27.3 ± 4.7	26.9 ± 5.1	27.8 ± 4.2
Smoking status			
Never	157,723 (54.6)	92,834 (59.6)	64,889 (48.7)
Previous	103,171 (35.7)	49,887 (32)	53,284 (40)
Current	28,107 (9.7)	13,090 (8.4)	15,017 (11.3)
Drinking status			
Never	8,843 (3.1)	6,649 (4.3)	2,194 (1.7)
Previous	9,194 (3.2)	5,191 (3.3)	4,003 (3)
Current	270,964 (93.8)	143,971 (92.4)	126,993 (95.4)
eBMD, g/cm^2^	0.5468 ± 0.1353	0.5221 ± 0.1186	0.5755 ± 0.1472
DXA-BMD, g/cm^2^	1.2026 ± 0.1498	1.1164 ± 0.1227	1.2945 ± 0.1180
Townsend deprivation index	−1.7 ± 2.9	−1.7 ± 2.8	−1.6 ± 2.9
White blood cells (10^9^/L)	6.88 ± 2.17	6.85 ± 2.12	6.91 ± 2.22
Neutrophils (10^9^/L)	4.23 ± 1.40	4.20 ± 1.39	4.27 ± 1.42
Lymphocytes (10^9^/L)	1.95 ± 1.21	2.00 ± 1.03	1.89 ± 1.38
Monocytes (10^9^/L)	0.48 ± 0.31	0.44 ± 0.36	0.52 ± 0.22
CRP (mg/L)	2.55 ± 4.35	2.64 ± 4.32	2.43 ± 4.38
Vitamin D, nmol/L	49.9 ± 20.9	49.9 ± 20.9	50.0 ± 21.0
METs minutes/week	2,655 ± 2,451	2,544 ± 2,188	2,785 ± 2,720
Hip fracture			
Yes	2,655 (0.9)	1,749 (1.1)	906 (0.7)
Fracture			
Yes	16,121 (5.6)	10,269 (6.6)	5,852 (4.4)
Fall			
Yes	12,967 (4.5)	7,601 (4.9)	5,366 (4.0)
Chronotype			
Low risk	182,964 (63.3)	99,546 (63.9)	83,418 (62.6)
High risk	106,037 (36.7)	56,265 (36.1)	49,772 (37.4)
Duration			
Low risk	200,482 (69.4)	108,516 (69.7)	91,966 (69.1)
High risk	88,519 (30.6)	47,295 (30.4)	41,224 (31)
Insomnia			
Low risk	209,109 (72.4)	107,101 (68.7)	102,008 (76.6)
High risk	79,892 (27.6)	48,710 (31.3)	31,182 (23.4)
Snoring			
Low risk	181,222 (62.7)	112,117 (72)	69,105 (51.9)
High risk	107,779 (37.3)	43,694 (28)	64,085 (48.1)
Sleepiness			
Low risk	281,787 (97.5)	152,328 (97.8)	129,459 (97.2)
High risk	7,214 (2.5)	3,483 (2.2)	3,731 (2.8)
Health sleep score*			
0	408 (0.1)	175 (0.1)	233 (0.2)
1	6,081 (2.1)	3,067 (2)	3,014 (2.3)
2	31,496 (10.9)	16,133 (10.4)	15,363 (11.5)
3	80,640 (27.9)	40,674 (26.1)	39,966 (30)
4	107,309 (37.1)	56,557 (36.3)	50,752 (38.1)
5	63,067 (21.8)	39,205 (25.2)	23,862 (17.9)

*A higher score indicates a higher healthy sleep pattern.

In the multivariable models, a greater healthy sleep score was associated with lower risks of incident falls (*p*-value for trend = 2.40E-10), all fractures (*p*-value for trend = 1.00E-23), and hip fracture (*p*-value for trend = 5.20E-05). In contrast to participants with an unfavorable sleep pattern, those with a healthy sleep pattern experienced a 17% and 28% reduced risk of incident falls (HR, 0.83; 95% CI, 0.74–0.93) and all fractures (HR, 0.72; 95% CI, 0.66–0.79), respectively, during follow-up (**Tables 2**, **3**). We find no significant difference between the various sleep patterns for developing hip fracture. In addition, the low-risk groups of the individual sleep behaviors included in the healthy sleep score, except snoring, exhibited significantly lower risks of falls and all fractures. For hip fracture, significant associations were found for sleep duration, chronotype, and sleepiness. The sensitivity analysis of the health sleep score excluding snoring showed similar associations with the risks of falls and fractures.

**Table 2:** Association between sleep factors and incident falls and fractures.

Sleep factors*	All		Women		Men		*p*_interaction_
	HR, 95% CI	*p*	HR, 95% CI	*p*	HR, 95% CI	*p*	
Fall							
Sleep score	0.95 (0.93, 0.96)	2.40E-10	0.94 (0.92, 0.96)	2.30E-08	0.95 (0.93, 0.98)	9.40E-04	8.90E-01
Chronotype	0.94 (0.91, 0.97)	6.60E-04	0.92 (0.88, 0.97)	1.10E-03	0.96 (0.91, 1.01)	1.40E-01	4.40E-01
Sleep duration	0.89 (0.86, 0.92)	3.60E-10	0.90 (0.86, 0.95)	3.30E-05	0.88 (0.83, 0.93)	4.30E-06	6.60E-02
Insomnia	0.92 (0.88, 0.95)	1.10E-05	0.92 (0.88, 0.96)	6.30E-04	0.92 (0.86, 0.98)	6.20E-03	5.90E-01
Sleepiness	0.83 (0.75, 0.92)	5.30E-04	0.86 (0.74, 0.99)	3.60E-02	0.82 (0.71, 0.95)	9.20E-03	3.50E-01
Snoring	1.03 (0.99, 1.07)	1.00E-01	0.99 (0.94, 1.04)	6.20E-01	1.07 (1.01, 1.13)	1.50E-02	1.20E-01
All fractures							
Sleep score	0.92 (0.91, 0.94)	1.00E-23	0.92 (0.91, 0.94)	2.30E-16	0.93 (0.91, 0.95)	3.60E-08	7.70E-01
Chronotype	0.94 (0.91, 0.97)	1.50E-04	0.93 (0.89, 0.97)	3.10E-04	0.96 (0.91, 1.02)	1.80E-01	7.80E-01
Sleep duration	0.88 (0.86, 0.91)	1.30E-13	0.90 (0.86, 0.94)	2.50E-07	0.87 (0.83, 0.92)	7.40E-07	2.10E-01
Insomnia	0.86 (0.83, 0.89)	1.30E-18	0.87 (0.84, 0.91)	3.10E-11	0.84 (0.80, 0.89)	5.50E-09	4.60E-01
Sleepiness	0.80 (0.73, 0.87)	2.40E-07	0.82 (0.73, 0.92)	6.00E-04	0.76 (0.67, 0.87)	5.40E-05	4.00E-01
Snoring	1.00 (0.97, 1.04)	9.30E-01	0.97 (0.93, 1.02)	2.50E-01	1.04 (0.99, 1.09)	1.50E-01	1.40E-01
Hip fracture							
Sleep score	0.93 (0.89, 0.96)	5.20E-05	0.92 (0.87, 0.96)	1.70E-04	0.94 (0.88, 1.01)	8.70E-02	4.80E-01
Chronotype	0.90 (0.83, 0.98)	9.80E-03	0.92 (0.83, 1.01)	7.90E-02	0.88 (0.77, 1.01)	7.40E-02	4.60E-01
Sleep duration	0.83 (0.76, 0.89)	2.30E-06	0.81 (0.74, 0.90)	3.70E-05	0.86 (0.75, 0.99)	3.00E-02	6.90E-01
Insomnia	0.93 (0.85, 1.01)	6.80E-02	0.92 (0.84, 1.02)	1.10E-01	0.93 (0.80, 1.08)	3.40E-01	8.40E-01
Sleepiness	0.73 (0.60, 0.90)	2.40E-03	0.65 (0.51, 0.84)	7.20E-04	0.87 (0.61, 1.22)	4.20E-01	1.70E-01
Snoring	1.07 (0.98, 1.16)	1.20E-01	1.03 (0.93, 1.15)	5.60E-01	1.11 (0.97, 1.27)	1.40E-01	2.40E-01

Data were adjusted for age, sex, BMI, assessment center, deprivation status, smoking status (never, previous, or current), alcohol intake status (never, previous, or current), physical activity (MET-minutes/week), and serum vitamin D level.

*Healthy sleep factor. HR is obtained by comparing the low- vs. high-risk group of these factors.

**Table 3:** Risk of incident falls and fractures according to the healthy sleep patterns*.

	Healthy Sleep Patterns		
	Favorable (score 4–5)	Intermediate (score 2–3)	Unfavorable (score 0–1)
*N*	170,376	112,135	6,489
Falls			
No. of falls	7,405	5,253	309
HR (95% CI)	0.83 (0.74, 0.93)	0.91 (0.81, 1.02)	1 [Reference]
* p*-value	1.5E-03	1.0E-01	
* p*-value for trend	2.40E-10		
All fractures			
No. of all fractures	8,922	6,720	479
HR (95% CI)	0.72 (0.66, 0.79)	0.82 (0.75, 0.90)	1 [Reference]
* p*-value	5.6E-12	3.4E-05	
* p*-value for trend	1.00E-23		
Hip fracture			
No. of hip fracture	1,452	1,135	68
HR (95% CI)	0.81 (0.63, 1.03)	0.95 (0.75, 1.22)	1 [Reference]
* p*-value	8.4E-02	7.0E-01	
* p*-value for trend	5.20E-05		

Data were adjusted for age, sex, BMI, assessment center, deprivation status, smoking status (never, previous, or current), alcohol intake status (never, previous, or current), physical activity (MET-minutes/week), and serum vitamin D level.

* The healthy sleep score is defined based on the health sleep score.

We further analyzed the associations between a healthy sleep pattern and BMD and found that participants with a healthy sleep pattern were associated with higher eBMD (β [SE] 0.011 [0.002], *p* = 2.7E-07) and DXA-BMD (β [SE] 0.018 [0.005], *p* = 8.6E-04) when compared with those with an unfavorable sleep pattern after full adjustment (**Table 4**).

**Table 4:** Associations between sleep factors and BMD.

Sleep factors*	All		Women		Men		*p*_interaction_
	β (SE)	*p*	β (SE)	*p*	β (SE)	*p*	
eBMD							
Sleep score	0.0039 (0.0003)	7.70E-34	0.0031 (0.0004)	1.80E-15	0.0043 (0.0005)	9.60E-16	8.60E-01
Chronotype	0.0042 (0.0007)	2.80E-10	0.0039 (0.0008)	1.60E-06	0.0034 (0.0011)	1.70E-03	2.40E-03
Sleep duration	0.0049 (0.0007)	1.50E-12	0.0026 (0.0008)	2.10E-03	0.0064 (0.0011)	1.50E-08	2.40E-01
Insomnia	0.0039 (0.0007)	7.30E-08	0.0021 (0.0008)	9.70E-03	0.0055 (0.0012)	9.00E-06	7.00E-01
Sleepiness	0.0037 (0.0021)	7.00E-02	0.0089 (0.0026)	6.80E-04	0.0013 (0.0032)	6.80E-01	1.30E-03
Snoring	0.0045 (0.0007)	4.70E-11	0.0050 (0.0009)	7.80E-09	0.0039 (0.0011)	2.40E-04	5.90E-04
BMD-DXA							
Sleep score	0.0037 (0.0007)	2.10E-07	0.0027 (0.0010)	5.20E-03	0.0037 (0.0010)	3.60E-04	5.60E-01
Chronotype	−0.0003 (0.0014)	8.50E-01	−0.0020 (0.0020)	3.00E-01	0.0008 (0.0021)	6.90E-01	7.90E-04
Sleep duration	0.0080 (0.0016)	3.30E-07	0.0063 (0.0021)	2.90E-03	0.0080 (0.0022)	4.10E-04	9.30E-01
Insomnia	0.0078 (0.0016)	1.40E-06	0.0054 (0.0021)	9.60E-03	0.0094 (0.0025)	1.40E-04	9.30E-01
Sleepiness	−0.0002 (0.0048)	9.70E-01	0.0041 (0.0069)	5.50E-01	−0.0005 (0.0067)	9.40E-01	3.00E-01
Snoring	0.0025 (0.0015)	9.30E-02	0.0033 (0.0022)	1.40E-01	0.0006 (0.0020)	7.90E-01	7.50E-02

Data were adjusted for age, sex, assessment center, BMI, deprivation status, physical activity (MET-minutes/week), smoking status (never, previous, or current), alcohol intake (never, previous, or current), and serum vitamin D level.

*Healthy sleep factors.

β is obtained by comparing the low- vs. high-risk group of these factors.

We also performed sex-stratified analyses for the association between the healthy sleep score and these bone health-related outcomes, and significant protective associations were consistently observed for all three outcomes among women and men (all *p* < 0.05), except for hip fracture among men. No interaction was observed for the associations of the healthy sleep score and sex with incident falls, all fractures, and hip fracture (**Table 3**). Immune parameters, including white blood cells, neutrophils, monocytes, lymphocytes, and CRP, did not significantly modify the association between sleep and BMD, falls, and fractures. Further mediation analysis showed that circulating CRP mediated a small but significant proportion (1%) of the total effect of sleep score on eBMD, whereas other parameters mediated no or neglectable proportions.

**Figure 1:**
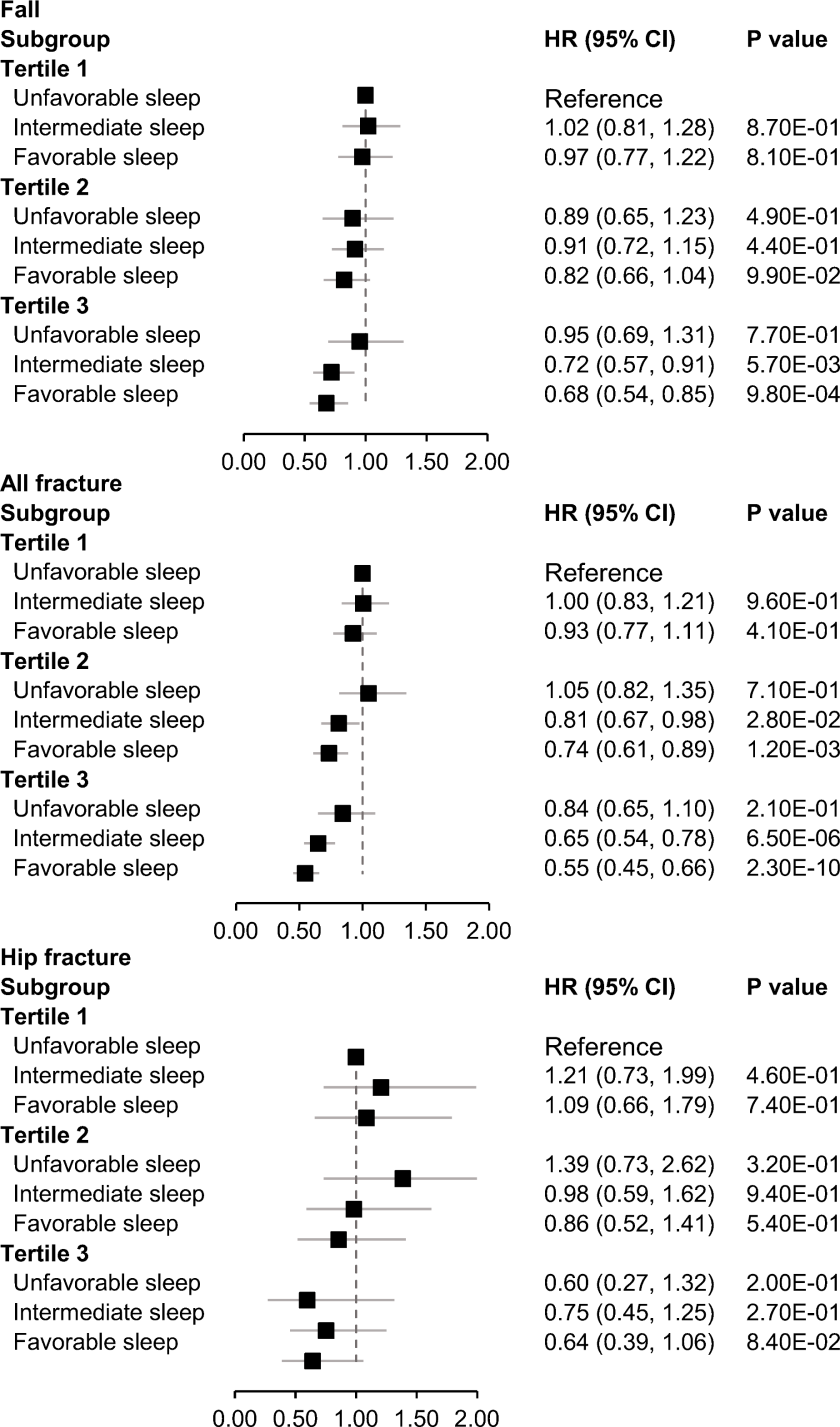
Joint association of genetically determined BMD and sleep patterns with incident falls and fractures. Data were adjusted for age, sex, BMI, assessment center, deprivation status, smoking status (never, previous, or current), alcohol intake status (never, previous, or current), physical activity (MET-minutes/week), and serum vitamin D level. PGS, polygenic scores. A higher PGS indicates a higher genetically determined BMD. Low, intermediate, and high groups of the PGS were categorized according to tertiles of the PGS.

**Figure 2:**
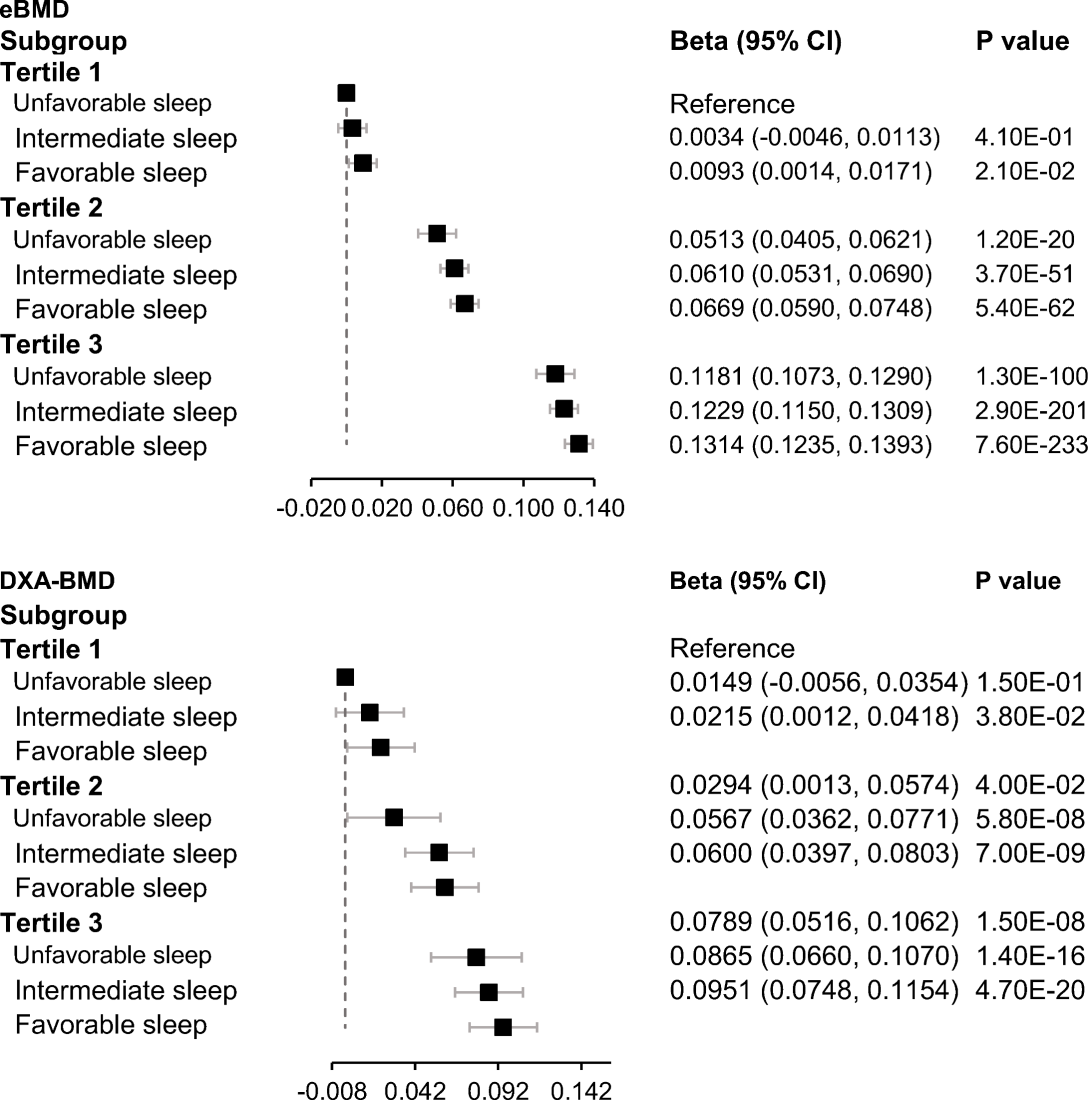
Joint association of genetically determined BMD and sleep patterns with BMD. Data were adjusted for age, sex, BMI, assessment center, deprivation status, smoking status (never, previous, or current), alcohol intake status (never, previous, or current), physical activity (MET-minutes/week), serum vitamin D level, genetic batch, and the first 10 principal components. PGS, polygenic scores. A higher PGS indicates a higher genetically determined BMD. Low, intermediate, and high groups of the PGS were categorized according to tertiles of the PGS.

We next jointly analyzed genetically determined BMD and sleep patterns. Even though no significant interaction was observed between genetically determined BMD and the sleep patterns on falls, all fractures, and hip fracture, participants with a healthy sleep pattern and high genetically determined BMD appeared to have the lowest risks of falls and fractures (**Figures 1**, **2**).

In the sensitivity analyses, after adjusting for menopausal status, the association slightly decreased, but most of the previously significant results were maintained. A smaller sample size and its potential influence on bone health may partly account for this attenuation (**Supplementary Tables 2, 3**). In addition, the results were fundamentally the same after excluding incident cases during the initial 2 years of follow-up in the sensitivity analysis.

## Discussion

In the current study, a 19% and 27% decrease in the risk of developing incident and all fractures, respectively, during follow-up were observed in participants who adhered to a healthy sleep pattern, compared to those who did not. The favorable associations between the healthy sleep pattern and incident falls and fractures were similar among women and men. Joint association analysis showed that participants exhibiting both a healthy sleep pattern and a higher genetically determined BMD experienced the lowest risks of falls and fractures.

Our findings are in line with previous studies showing that abnormal sleep duration (16), sleepiness, insomnia (7), and snoring (25) were associated with higher risks of falls and fractures (13, 26–28). For example, data from the Women’s Health Initiative showed that short sleep duration was related to reduced BMD, along with a higher risk of osteoporosis and an increased fracture risk (6, 8). Another study consisting of 8,101 women (mean age, 77.0 years) reported that daily napping was related to risks of fractures (13). An observational meta-analysis suggested that both insufficient and excessive sleep duration were linked to a higher risk of falls (26).

Of note, the past investigations only examined the sleep behaviors individually, ignoring the collective sleep pattern that comprised various highly correlated sleep behaviors (19). For example, longer sleep duration may be indicative of sleep-disordered breathing and may result in excessive daytime sleepiness, which could potentially exacerbate the risks of falls and fractures (13). For the first time, we reported the positive associations of a healthy sleep pattern with reduced risks of falls and fractures. The use of a healthy sleep pattern provides an accurate and comprehensive description of sleep behavior and facilitates the translation of our findings into specific recommendations for sleep behaviors.

Our findings indicate that different sleep behaviors appeared to have an additive impact on bone health, and several plausible mechanisms may account for this association. Sleep may affect bone through immune factors. Sleep plays a crucial role in supporting the immune system. Sleep disorders have been linked to changes in both innate and adaptive immune responses, resulting in a persistent inflammatory condition and an elevated susceptibility to autoimmune diseases (29). Evidence suggests that pathogenic bone loss is associated with immune disruption caused by the presence of inflammatory markers and autoantibodies (30). This mechanism is supported by our finding that CRP serves as a mediator in the pathway between sleep and bone health. Sleep disorders also disrupt the circadian rhythm (31), hormone regulation, and sympathetic activation, and involve metabolic derangement (6, 32, 33). Sleep deprivation decreases the levels of leptin (34), which may protect from bone loss by limiting excessive bone resorption (35). An intervention trial suggested that 3 weeks of circadian disruption accompanied by concurrent sleep decreased bone formation but had no effect on bone resorption (36). Data from an animal study suggested that chronic sleep deprivation decreased BMD, 25(OH)D, and osteogenesis, and impaired mineralization (37).

Women are more likely to have higher risks of falls (38) and fractures (39) than men, partly because women tend to have more bone loss in BMD along with aging (40). However, our study did not yield a significant difference in the association between a healthy sleep pattern and risks of falls and fractures among men and women. This finding suggests that a recommendation for following a healthy sleep pattern is equally important among men and women when designing falls and fractures prevention programs.

Currently, no prior study has examined the association between the combination of healthy sleep behaviors and genetic risk factors and incident falls and fractures. Though we did not find a statistically significant interaction between healthy sleep patterns and PGS of BMD on bone health, our finding suggests that a healthy sleep pattern and high genetically determined BMD may promote bone health in an additive manner. These results also indicate that a healthy sleep pattern is essential to promote bone health regardless of the individuals’ genetic background of BMD.

## Strength and limitations

To our best knowledge, for the first time, we examined the association of sleep patterns and its combination with genetically determined BMD with falls and fractures. Moreover, the substantial size of the study population and the comprehensive data on covariates allowed for a robust analysis, effectively controlling for numerous established and potential confounders associated with bone health. To minimize the risk of reverse causation, we intentionally removed individuals with a history of fracture at the beginning of the study. However, several limitations of our study also merit consideration. First, unmeasured confounding and reverse causation might exist. Sleep data were self-reported, which may introduce misclassification. However, misclassification errors are likely to have biased these findings toward the null. Second, we only studied the association of a healthy sleep pattern in elder white British populations. As a result, the possibility of generalizing our findings to diverse ethnic groups or different age groups may be limited. Third, the utility of the score might be influenced by its subjectivity and its narrow focus on specific parameters. Last, falls and fractures are not always the result of a single cause. Given that falls and fractures are heterogeneous and complex traits, we cannot differentiate the driver of bone loss from other causes, including weakened muscle strength and poor physical function.

## Conclusion

In summary, a healthy sleep pattern was significantly related to decreased risks of falls and fractures. This association was similar among all genetically determined BMD categories. The study points to the potential advantages of following a healthy sleep pattern for preventing falls and fractures.

## Data availability statement

Publicly available datasets were analyzed in this study. This data can be found here: the UK Biobank Resource under Application Number 29256.

## Ethics statement

The studies involving humans were approved by the North West Multi-Center Research Ethics Committee (REC reference: 11/NW/03820). The studies were conducted in accordance with the local legislation and institutional requirements. Written informed consent for participation in this study was provided by the participants’ legal guardians/next of kin. Written informed consent was obtained from the individual(s) for the publication of any potentially identifiable images or data included in this article.

## Author contributions

LQ had complete access to all of the data in the study and assumes responsibility for the integrity and the accuracy of the data analysis. Concept and design: TZ, LQ. Acquisition, analysis, or interpretation of data: TZ, LQ, XD, YY, QX, and YH. Drafting of the manuscript: TZ, LQ, and YY. Critical revision of the manuscript for important intellectual content: All authors. Statistical analysis: LQ, TZ, and YY. Obtained funding: LQ. Study supervision: LQ. All authors contributed to the article and approved the submitted version.
