# Supplementary material for: Adherence to a healthy sleep pattern is associated with lower risks of incident falls and fractures during aging
Source: Front Immunol. 2023 Aug 17;14:1234102. doi: 10.3389/fimmu.2023.1234102 (PMC10470625; doi:10.3389/fimmu.2023.1234102)
Supplement: Supplementary file 1 [file Table_1.docx]

| **Supplementary Table 1** List of ICD10 codes used to define fracture cases (1) | |  |  |
| --- | --- | --- | --- |
| **Fracture Site** | **Fragility Fracture Codes** | **Pathological Fracture* Codes** | **Stress Fracture Codes** |
| Forearm, distal end | S52.5, S52.6, S62.9 |  |  |
| Forearm, forearm shaft | S52.2-S52.4 |  |  |
| Forearm, proximal end | S52.0, S52.1 |  |  |
| Forearm, other forearm fractures | S52.8, S52.9, M8003, M8083 | M8443 | M8433 |
| Upper arm, proximal humerus | S42.2, S42.3 | M8441, M8442 | M8431, M8432 |
| Hip | S72.0-S72.9 | M84459 | M84359 |
| Vertebrae | S12.0-S12.6, S12.7, S22.0, S22.1, S32.0, M484, M485, M8008, M8088 |  |  |
| Upper arm, distal end of humerus | S42.4 |  |  |
| Upper arm, other fractures of upper arm | S42.7-S42.9, M8001, M8002, M8081, M8082 |  |  |
| Pelvis | S32.1-S32.9 | M84454 | M84350 |
| Femur | S71.1, S72.3-S72.9, M8005, M8085 | M84451-M84453 | M84351-M84353 |
| Lower leg | S82.1-S82.4, M8006, M8086 | M8446 | M8436 |
| Patella | S82.0 |  |  |
| Ankle | S82.5-S82.9, M8007, M8087 | M84471-M84473 | M84371-M84373 |
| Rib | S22.3-S22.5 |  |  |
| Scapula | S42.1 |  |  |
| Sternum | S22.2 |  |  |
| Clavicle | S42.0 |  |  |
| Fracture site unspecified | M8000, M8080, M810, Z87310 | Z87311 |  |

*Not specified as osteoporotic neoplastic, or as other disease

(1) Morris JA, Kemp JP, Youlten SE, Laurent L, Logan JG, Chai RC, Vulpescu NA, Forgetta V, Kleinman A, Mohanty ST, et al. An atlas of genetic influences on osteoporosis in humans and mice. *Nature Genetics* (2019) 51:258–266. doi: 10.1038/s41588-018-0302-x

**Supplementary Table 2** Associations between sleep factors and BMD among women

| Sleep factors* | Women | |
| --- | --- | --- |
|  | β (SE) | *P* |
| **eBMD** |  |  |
| Sleep score | 0.0026 (0.0004) | 7.00E-10 |
| Chronotype | 0.0038 (0.0009) | 1.30E-05 |
| Sleep duration | 0.0023 (0.0009) | 1.30E-02 |
| Insomnia | 0.0010 (0.0009) | 2.60E-01 |
| Sleepiness | 0.0078 (0.0029) | 6.80E-03 |
| Snoring | 0.0043 (0.0009) | 6.30E-06 |
| **BMD-DXA** |  |  |
| Sleep score | 0.0021 (0.0010) | 4.90E-02 |
| Chronotype | -0.0016 (0.0021) | 4.50E-01 |
| Sleep duration | 0.0063 (0.0023) | 6.00E-03 |
| Insomnia | 0.0014 (0.0023) | 5.30E-01 |
| Sleepiness | 0.0041 (0.0074) | 5.80E-01 |
| Snoring | 0.0039 (0.0024) | 1.00E-01 |

Data were adjusted for age, sex, assessment center, BMI, deprivation status, physical activity (MET-minutes/week), smoking status (never, previous, current), alcohol intake (never, previous, current), serum vitamin D level, and menopause status.

**Supplementary Table 3** Association between sleep factors and incident falls and fractures among women

| Sleep factors* | Women | |
| --- | --- | --- |
|  | HR, 95% CI | *P* |
| **Fall** |  |  |
| Sleep score | 0.95 (0.93, 0.97) | 2.80E-05 |
| Chronotype | 0.92 (0.87, 0.97) | 9.60E-04 |
| Sleep duration | 0.93 (0.88, 0.98) | 5.60E-03 |
| Insomnia | 0.94 (0.89, 0.99) | 2.30E-02 |
| Sleepiness | 0.88 (0.75, 1.03) | 1.20E-01 |
| Snoring | 1.00 (0.94, 1.05) | 9.20E-01 |
| **All fractures** |  |  |
| Sleep score | 0.93 (0.91, 0.95) | 2.60E-12 |
| Chronotype | 0.93 (0.89, 0.97) | 1.10E-03 |
| Sleep duration | 0.91 (0.87, 0.95) | 5.70E-05 |
| Insomnia | 0.87 (0.84, 0.91) | 4.40E-09 |
| Sleepiness | 0.81 (0.71, 0.92) | 1.20E-03 |
| Snoring | 0.98 (0.93, 1.03) | 4.00E-01 |
| **Hip fracture** |  |  |
| Sleep score | 0.93 (0.88, 0.97) | 2.30E-03 |
| Chronotype | 0.89 (0.80, 0.99) | 3.90E-02 |
| Sleep duration | 0.84 (0.75, 0.93) | 9.30E-04 |
| Insomnia | 0.94 (0.84, 1.05) | 2.70E-01 |
| Sleepiness | 0.66 (0.50, 0.86) | 2.50E-03 |
| Snoring | 1.06 (0.95, 1.20) | 3.00E-01 |
| Data were adjusted for age, sex, assessment center, BMI, deprivation status, physical activity (MET-minutes/week), smoking status (never, previous, current), alcohol intake (never, previous, current), serum vitamin D level, and menopause status. | | |
| *Healthy sleep factor. The HR is obtained by comparing the low v.s. high risk group of these factors. | | |
